# *Cutibacterium* Adaptation to Life on Humans Provides a Potential *C acnes* Infection Biomarker

**DOI:** 10.1016/j.jid.2025.03.048

**Published:** 2025-05-26

**Authors:** Md Shafiuddin, Wen-chi Huang, Gabriel William Prather, Jeffrey Ryan Anton, Andrew Lawrence Martin, Sydney Brianna Sillart, Jonathan Z. Tang, Michael R. Vittori, Michael J. Prinsen, Jessica Jane Ninneman, Chandrashekhara Manithody, Jeffrey P. Henderson, Alexander W. Aleem, Ma Xenia Garcia Ilagan, William H. McCoy

**Affiliations:** 1 Division of Dermatology, Department of Medicine, Washington University School of Medicine in St. Louis, St. Louis, Missouri, USA; 2 Division of Biology & Biomedical Sciences, Washington University School of Medicine in St. Louis, St. Louis, Missouri, USA; 3 Washington University School of Medicine in St. Louis, St. Louis, Missouri, USA; 4 High-Throughput Screening Center, Center for Drug Discovery, Department of Biochemistry and Molecular Biophysics, Washington University School of Medicine in St. Louis, St. Louis, Missouri, USA; 5 Division of Infectious Diseases, Department of Medicine, Washington University School of Medicine in St. Louis, St. Louis, Missouri, USA; 6 Department of Orthopedic Surgery, Washington University School of Medicine in St. Louis, St. Louis, Missouri, USA

**Keywords:** Acne, Heme, *Propionibacterium*, Prosthesis, RoxP

## Abstract

Propionibacteriaceae appear to have adapted to life on humans during the domestication of cattle. These microbial immigrants formed the genus *Cutibacterium,* and a descendent of those microbial trailblazers (*C acnes)* now dominates 25% of human skin. *C acnes* colonization of human skin requires the protein RoxP. Although all *Cutibacteria* encode this adaptation to life on humans, nothing like RoxP has been found in any other organism. In this study, we report an extensive assessment of 21 RoxP orthologs, which identified conserved molecular surfaces linked to heme-dependent oligomerization and low pH stability. Our investigation suggests how RoxP helps *C acnes* dominate sebaceous skin, and it identified an ortholog associated with the emergence of an acne vulgaris–associated, pathobiont subspecies. *C acnes* is also an emerging pathogen that frequently infects joint prostheses and other medical devices. These infections are often missed, because there is no test to confirm a *C acnes* infection. To address this clinical need, we developed immunoassays that can assess RoxP in human biofluids commonly infected by *C acnes.* This study’s findings and assays will help shed light on the consequences of Neolithic Age livestock domestication, the evolution of skin commensals into pathogens, and how to identify infections of human “replacement parts.”

## INTRODUCTION

Propionibacteriaceae grow in numerous environments (eg, animals, water, sewage, soil) (Stackebrandt, 2014). When humans domesticated cows, it appeared that a bovine Propionibacteriaceae family member adapted to human skin (Scholz and Kilian, 2016). *Cutibacterium* (formerly known as *Propionibacterium* [Scholz and Kilian, 2016]) *acnes* is the progeny of that microbial immigrant, and it evolved to dominate 25% of adult human skin. *C acnes* is a lipophilic, gram-positive, aerotolerant anaerobe that accounts for 92% of the microbes that live on human sebaceous skin (Conwill et al, 2022; Oh et al, 2014) (eg, head, neck, upper trunk). This skin contains a high density of pilosebaceous units (PSUs) whose sebaceous glands produce sebum, a lipid-rich secretion that moisturizes human skin (Makrantonaki et al, 2011; Shelmire, 1959). *C acnes* catabolizes sebum triglycerides forming free fatty acids (Puhvel et al, 1975), and it significantly contributes to the low pH found on human skin (Marples et al, 1971). The major *C acnes* free fatty acid, propionic acid, impedes pathogen colonization (eg, *Staphylococcus aureus)* (Shu et al, 2013) while also improving skin barrier function by inducing epidermal lipid synthesis (Almoughrabie et al, 2023).

*C acnes* is part of the normal human skin flora (Fitz-Gibbon et al, 2013; Grice et al, 2009; McCoy et al, 2019), yet it also contributes to human disease. *C acnes* contributes to acne vulgaris (Agak et al, 2014), which impacts nearly every human (James, 2005). In the United States each year, acne incurs $1.3 billion dollars in healthcare costs (Lim et al, 2017) and leads to >2 million office visits (James, 2005). At these visits, patients are prescribed topical antimicrobial therapies and five million oral antibiotic prescriptions per year (Stern, 2000). Extensive prior exposure to these antimicrobial treatments likely explains in part why *C acnes* is not effectively reduced by presurgical skin sterilization or prophylactic antibiotics (Namdari et al, 2017; Rao et al, 2018; Saltzman et al, 2009). The effectiveness of these strategies to reduce surgical-site infections by other bacteria (eg, *Staphylococci)* has likely contributed to *C acnes* becoming an emerging pathogen of indwelling medical devices (IMDs) inserted through human skin (Achermann et al, 2014).

*C acnes’* survival in the face of these measures may in part stem from its propensity to form biofilms (Bayston et al, 2007a, 2007b; Coenye et al, 2007; Qi et al, 2008; Ramage et al, 2003; Tunney et al, 2007). *C acnes* have been observed as macrocolonies that are consistent with biofilms both in its native PSU microenvironment (Jahns et al, 2012) and on medical devices, such as cerebrospinal fluid (CSF) shunt catheters (Bayston et al, 2007a) and hip prostheses (Tunney et al, 1999). With infection prophylaxis significantly reducing the burden of other microbes (eg, *Staphylococci*), *C acnes* has emerged as the most common cause of shoulder prosthesis infection (Frangiamore et al, 2017). *C acnes* is also tied with *S aureus* as the most common cause of sternal wound infections (Sandström et al, 2021), and it causes 4–15% of CSF shunt infections (Arnell et al, 2008; Hawkes et al, 2023; Sedano et al, 2023). Furthermore, the sonication of hip prostheses to dislodge microbial biofilms suggests that *C acnes* is the most common cause (62%) of hip periprosthetic joint infection (PJI) (Tunney et al, 1998).

*C acnes* IMD infection treatment normally requires one or more revision surgeries and long-term antibiotics (Paxton et al, 2019). This approach exacts a significant toll on a patient’s quality of life (QOL), and it is very costly. For shoulder *C acnes* PJI alone, these costs are projected to be >$250 million per year in the United States by 2030 (Baghdadi et al, 2017; Floyd et al, 2018; Padegimas et al, 2015). Unfortunately, some patients also succumb to their *C acnes* infections, as occurs for 15–27% of patients with *C acnes* endocarditis (Delahaye et al, 2005; Sohail etal, 2009). Significant contributors to these poor outcomes and high costs are routine delays in the identification of true *C acnes* infections. These delays are due to *C acnes*’ slow anaerobic growth (5–14 days) (Ellsworth et al, 2020) and ubiquitous nature.

During routine clinical microbiology cultures, *C acnes* is frequently dismissed as an environmental contaminant, particularly when more than one organism is isolated and the alternative is a microbe more commonly associated with infection (eg, *Staphylococci).* Yet, polymicrobial infections of coagulase-negative *Staphylococci* and *C acnes* have been reported (Frangiamore et al, 2017; Goldenberger et al, 2021; Sandstrom et al, 2021; Tunney et al, 1999, 1998), which suggests that culturing *Staphylococci* does not preclude *C acnes* contributing to an infection. Strategies are needed to help identify true *C acnes* infections. These strategies would allow for accurate assessments of *C acnes* infection incidence and the optimization of *C acnes* infection treatments. A better understanding of *C acnes* biology is necessary to develop these strategies. Genomic studies have revealed that *C acnes* lost 26 genes and acquired 108 genes as it moved from cows to humans (Scholz and Kilian, 2016), but those evolutionary adaptations have not been well-characterized.

*RoxP* (radical oxygenase of *Propionibacterium acnes)* is one of the genes that *Cutibacteria* acquired during their evolution to live on humans. Early RoxP work suggested that it may be an enzyme with antioxidant activity (Allhorn et al, 2016; Andersson et al, 2019), although later work has suggested that this antioxidant activity is nonspecific and due to nonenzymatic reduction of free radicals (Stødkilde et al, 2022). The *roxP* gene is only found in *Cutibacteria,* and it encodes a highly conserved, secreted, heme-binding protein (Allhorn et al, 2016). Since RoxP is often the most highly secreted protein of *C acnes* (Holland et al, 2010; Yu et al, 2016), it has been suggested that RoxP plays a significant functional role in host–microbe interactions. RoxP is essential for *C acnes* colonization of human skin ex vivo and survival under oxic environments (Allhorn et al, 2016). RoxP is remarkably conserved among all *C acnes* phylotypes, including several clinical isolates (Allhorn et al, 2016). A previous analysis of 106 *Cutibacterium* genomes showed that 86% of RoxP sequences share ≥99% identity, and 55% of RoxP sequences share 100% identity (Allhorn et al, 2016). Owing to its high degree of conservation among *Cutibacterium* genus members and its potential to counteract oxic environments, it is possible that RoxP played an important role in *C acnes* adaptation to human skin. Furthermore, it is a potential *Cutibacterium-specific* growth biomarker.

RoxP’s poorly understood role in *C acnes’* adaptation to life on humans led our group to perform an in-depth RoxP sequence analysis. This work led to biochemical investigations of several recombinant RoxP proteins, which demonstrated that ligand-dependent RoxP oligomerization is highly conserved in *Cutibacteria*. The high degree of RoxP sequence and functional conservation suggested to our group that anti-RoxP antibodies could help investigate RoxP’s role in *C acnes* biology. We then generated four anti-RoxP antibodies and used them to develop *Cutibacterium*-specific immunoassays that can assess RoxP secretion in culture medium and human biofluids.

## RESULTS

### Assessment of RoxP sequence space

**Figure 1.**
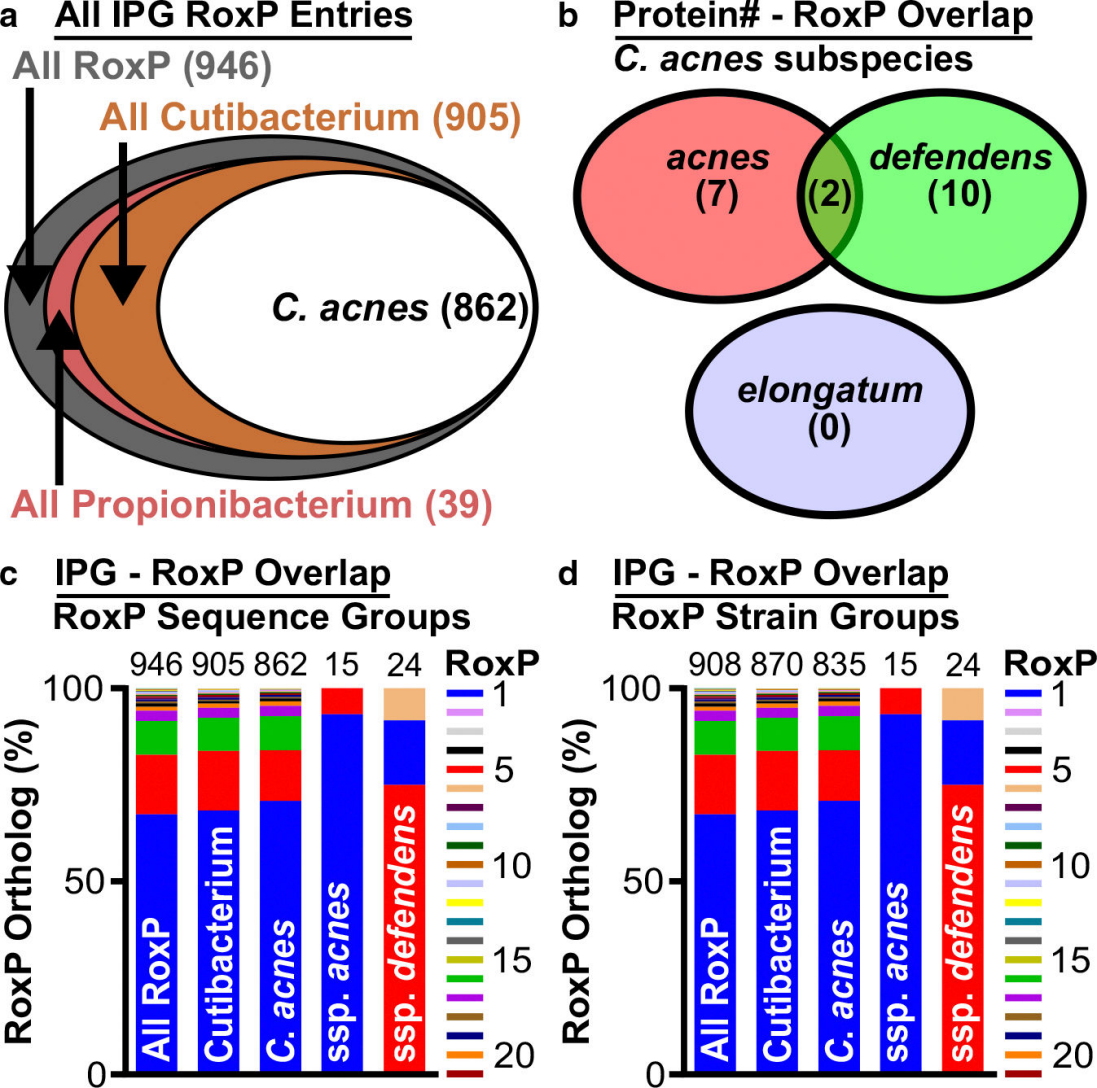
RoxP sequence space analysis. RoxP sequence space was assessed by identifying the IPG entries for each of the 21 RoxP orthologs and then assessing the number of entries shared at the (**a**) genus, (**b**, **c**) species, and (**d**) strain level. Species-level RoxP ortholog assessments were performed (**b**) first by direct comparison of the unique protein identification numbers listed for each IPG entry and (**c**) then by an assessment of IPG entries alone, which revealed that multiple “unique” entries in **b** collapsed to two orthologs (RoxP_1, 5) shared by *C acnes* subspecies *acnes* and *defendens* and one ortholog (RoxP_6) that is unique to *C acnes* subspecies *defendens.* (**c**) A comparison of *C acnes* subspecies *acnes* and *defendens* RoxP ortholog carriage also revealed subspecies-specific ortholog profiles (*C acnes* subspecies *acnes:* RoxP_1; *defendens:* RoxP_5). IPG, Identical Protein Group.

A BlastP search (Madden, 2002) using the RoxP protein sequence from *C acnes* strain KPA171202 (RoxP_1) identified 21 RoxP orthologs (RoxP_1–21, 71–99% identity to RoxP_1) (Supplementary Table S1). All sequence identities are relative to RoxP_1. RoxP_1 was chosen as the query sequence, because it was the first *roxP* gene evaluated (Allhorn et al, 2016). These RoxP ortholog sequences were used to query the National Center for Biotechnology Information Identical Protein Groups (IPG) to identify all available RoxP sequences. A total of 946 IPG RoxP entries were identified, and 91% were from *C acnes* (Figure 1a). Overlap in RoxP orthologs at the *C acnes* subspecies level was identified by assessing protein identification numbers (Figure 1b) and IPG entries (Figure 1c). The latter IPG assessment suggests that RoxP_6 is unique to *C acnes* subspecies *defendens*. RoxP_1 was found to be the most common RoxP ortholog among all RoxP sequences (67%), *Cutibacterium* spp (68%), *C acnes* (71%), and *C acnes* subspecies *acnes* (93%) (Figure 1c). Strain-level RoxP analysis was possible for 96% of entries and showed similar RoxP ortholog profiles to the entire RoxP sequence space (Figure 1c and d).

To expand upon this analysis, we then assessed *roxP* gene carriage in publicly available *Cutibacterium* genomes from named strains that included species-level information (n = 189). By assessing *roxP* gene carriage by each strain in the context of *C acnes* phylotypes (Supplementary Table S2), we determined that RoxP_1 is present in types I and II phylotypes, but it was not found in *C acnes* phylotype III. Other RoxP orthologs were found primarily in *C acnes* whole-genome sequence (WGS) clades IB-1 (RoxP_17, 99% identity), IB-2 (RoxP_16, 99% identity), II (RoxP_5, 82% identity; RoxP_6, 81% identity), and III (RoxP_2, 98% identity). RoxP_5 was also found in *C modestum* (formerly known as *Propionibacterium humerusii* [Goldenberger et al, 2021]), whereas RoxP_7 and _8 were exclusively found in *C namnetense.* This analysis demonstrated that some *Cutibacterium* species utilized specific RoxP orthologs, but it was not yet clear whether *C acnes* subspecies–specific RoxP orthologs existed beyond RoxP_6.

Although our initial assessment of RoxP sequence space did not identify a *C acnes* subspecies *elongatum* RoxP (Figure 1b and c), the use of phylotype III as a surrogate marker of this subspecies (Dekio et al, 2019) in Supplementary Table S2 entries that were only annotated as *C acnes* (n = 123) suggests that RoxP_2 is unique to *C acnes* subspecies *elongatum* (n = 3). Extending that strategy to *C acnes* subspecies *defendens* on the basis of phylotype II (McDowell et al, 2016) expanded our RoxP ortholog assessment of this subspecies from 5 annotated strains (80% RoxP_5, 20% RoxP_6) to 31 total strains (61% RoxP_5; 26% RoxP_1; 3% RoxP_4, 6, 18, 21). Applying this strategy to *C acnes* subspecies *acnes* (phylotype I) expanded our RoxP ortholog assessment of this subspecies from 2 annotated strains (100% RoxP_1) to 75 total strains (76% RoxP_1; 11% RoxP_16;7% RoxP_4;4% RoxP_17;1% RoxP_10, 19). IPG analysis also identified *C acnes* subspecies *acnes* with RoxP_5 (Figure 1c) (1 of 15 entries), though that was not observed in our genome analysis (0 of 75 strains). Subdividing this phylotype I search into IA/B/C revealed a similar ortholog profile for all three types (IA, n = 50: RoxP_1 > 16 > 17 > 19 [77 > 15 > 6 > 2%]; IB, n = 27: RoxP_1 > 16 > 17 > 10 [67 > 19 > 11 > 4%];IC, n = 1, RoxP_1). Interestingly, RoxP_17 appeared in strains that were labeled as IA/IB, suggesting that this ortholog may be unique to phylotype I strains that may be typed as IA or IB depending on the phylotyping strategy. This *roxP* gene carriage assessment clearly shows that there are specific RoxP ortholog profiles found in *C acnes* subspecies, and relatively, three orthologs segregate *C acnes* subspecies *acnes* (RoxP_1), *defendens* (RoxP_5, 6), and *elongatum* (RoxP_2).

The literature was then reviewed to determine which RoxP ortholog sequences had been previously experimentally evaluated (Supplementary Table S3). Although 43% of orthologs had been included in prior multiple sequence alignments (MSAs), there had been minimal evaluation of transcription (14%), secretion (29%), function (10%), or structure (5%). Analysis of the RoxP secretion literature (Supplementary Table S4) revealed that the secretion of six RoxP orthologs (RoxP_1, 2, 5, 16, 17, 19; sequence identity of 82–100%) had been previously observed, but only one of these studies directly investigated RoxP (Allhorn et al, 2016). All studies were performed under anoxic conditions at 37 °C, and most studies were performed during exponential growth phase in planktonic growth mode. All evaluated RoxP orthologs were found to be secreted into the media (cell-free). A subset of five orthologs (RoxP_1, 2, 5, 16, 17) was also found to be cell surface-associated (cell surface/wall) (Yu et al, 2016, 2015). This analysis of prior literature suggested that RoxP secretion and localization (cell-free, cell surface-associated) is conserved across orthologs.

### Assessment of RoxP sequence variation

**Figure 2.**
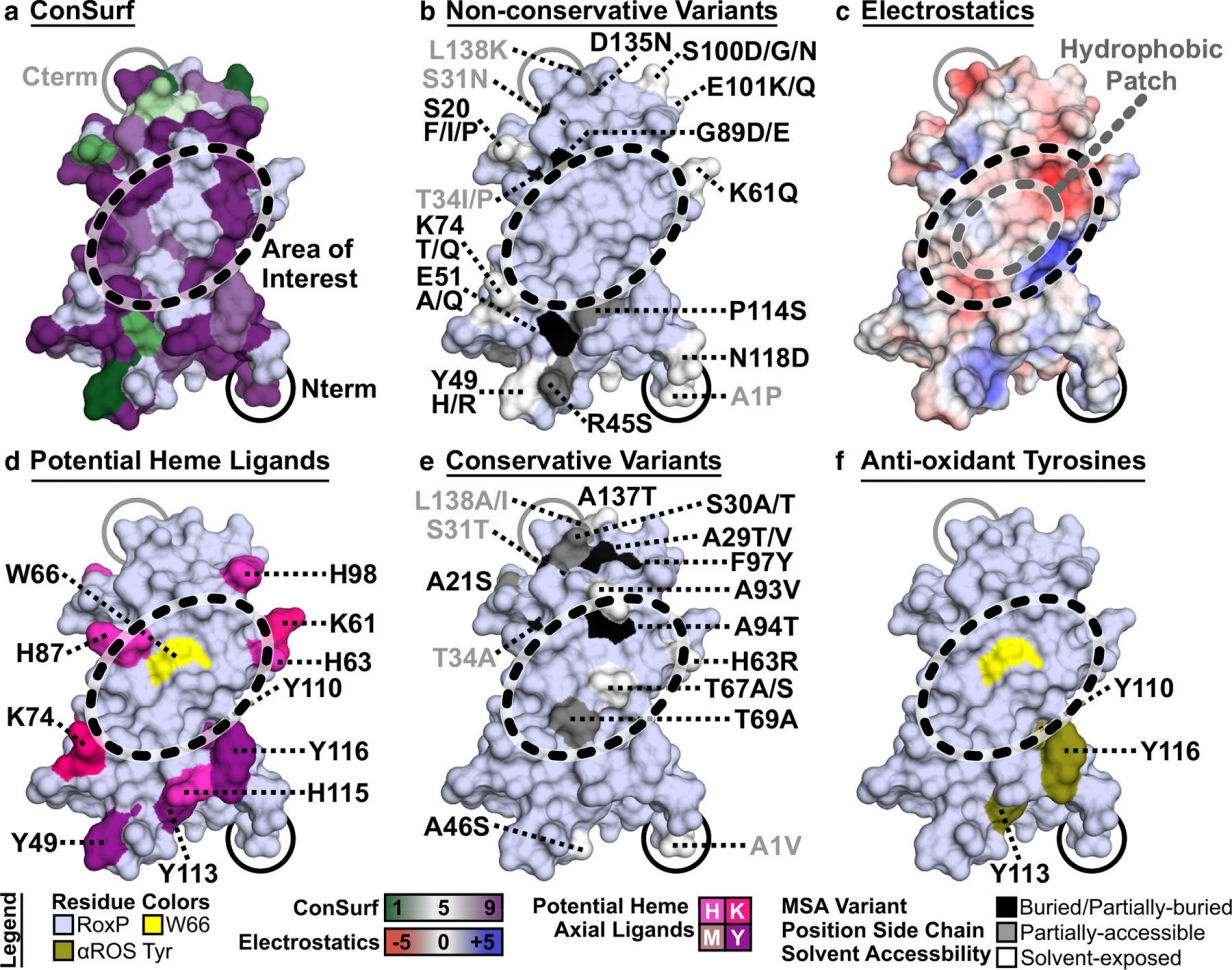
RoxP molecular surface conservation. RoxP molecular surface conservation, chemical nature, and amino acid exposure were evaluated using a RoxP surface generated in PyMOL (version2.5.2) (Schrödinger, 2022) from PDB 7bcj (NMR state 1). One RoxP molecular surface is shown inthis figure, with thearea of interest indicated by a black-dashed oval, whereas Supplementary Figure S1b–i includes this surface and four additional surface views. (**a**–**f**) The polypeptide termini (amino[N]-/carboxyl[C]-termini = N/Cterm) are indicated, and the Rox P surface was colored blue–white except where noted. (**a**, **b**, **e**) Surface conservation analysis is based on a MSAof21 RoxP orthologs (Supplementary FigureS1a). (**b**, **e**) Coloring of variable side chains based on solvent accessibility is the same as that described in Supplementary FigureS1aexceptthat buried and partially buried were combined. Buried (≤1%) residues did not contribute to any colored surface in these panels. (**a**) The ConSurf web server (http://consurf.tau.ac.il) used the RoxP MSA and PDB 7bcj to assess evolutionary conservation of amino acids (eg, low = 1, high = 9). The color of residue positions with insufficient variation to be assessed (eg, invariant positions) remained blue–white. (**b**, **e**) Residue positions with both nonconservative and conservative variants are labeled in gray. (**b**) Nonconservative variants (eg, a charge reversal such as Glu/E to Lys/K) are shown. Supplementary Material provides details fora complete definition of nonconservative/conservative substitutions and analysis of all RoxP residue variants. (**c**) An electrostatic surface was generated in PyMOL to identify positive (blue), uncharged (white), and negative (red) RoxP surfaces, which highlighted a hydrophobic patch (gray-dashed oval). (**d**) All potential heme axial ligands (Cys/C, His/H, Lys/K, Met/M, Tyr/Y) were colored shades of magenta, and the partially buried tryptophan (Trp66, W66) was colored yellow. RoxP’s two cysteines are not visible, because they form a buried, disulphide bond. (**e**) Conservative variants (eg, a small hydrophobic substitution such as Leu toVal) are shown. (**f**) All Tyr residues previously shown to be able to reduce free radicals (αROS: anti-reactive oxygen species [ROS]) were colored deep olive, and the partially buried W66 was colored yellow. MSA, multiple sequence alignment; NMR, nuclear magnetic resonance.

To explore RoxP sequence variation and attempt to correlate it with the prior literature, a MSA of all 21 RoxP orthologs was generated, and then the predicted per residue solvent-accessible surface area was calculated using the nuclear magnetic resonance (NMR) structure of apo-RoxP_1 (Stødkilde et al, 2022) (Supplementary Figure S1a and Supplementary Material). This information was used to evaluate conservation of molecular surface features such as amino acid side chains, electrostatics, and potential functional residues (Figure 2 and Supplementary Figure S1b–i). ConSurf analysis (Figure 2a) helped to identify a surface that excluded nonconservative amino acid variation (Figure 2b), contained a ~ 1200 Å^2^ hydrophobic patch (Figure 2c), and was surrounded by potential heme axial ligands (Figure 2d).

This surface also contained an invariant, partially surface-exposed (12.6% exposed compared with random coil) tryptophan (RoxP_1 W66) (Supplementary Figure S1). Surface-exposed tryptophan side chains have been reported to contribute to heme-binding (Samanta and Chakrabarti, 2001; Takahashi et al, 1984; Yammine et al, 2019). Evaluation of conservative amino acid variation (Figure 2e) also supported conservation of this unique interface with W66 at its center. This hydrophobic surface is distinct from the previously identified loop containing three invariant tyrosines (Y110, 113, 116) that mediate RoxP’s nonenzymatic, antioxidant activity (Figure 2f) (Stødkilde et al, 2022).

Conservation of this ~2000 Å^2^ surface suggests that it could be a conserved RoxP heme-binding site (HBS). A previous study demonstrated RoxP_1 heme-binding using Soret peak analysis, hemin-agarose pulldown, and NATIVE gel heme stain (Allhorn et al, 2016), whereas a subsequent study by the same group on RoxP_1 concluded that their Soret peak analysis did not demonstrate binding to either heme or protoporphyrin IX (PPIX) (Stødkilde et al, 2022). Owing to this inconsistency in experimental outcomes, we chose to conduct an in-depth biochemical analysis of RoxP ligand binding.

### Biochemical characterization of apo-RoxP

**Figure 3.**
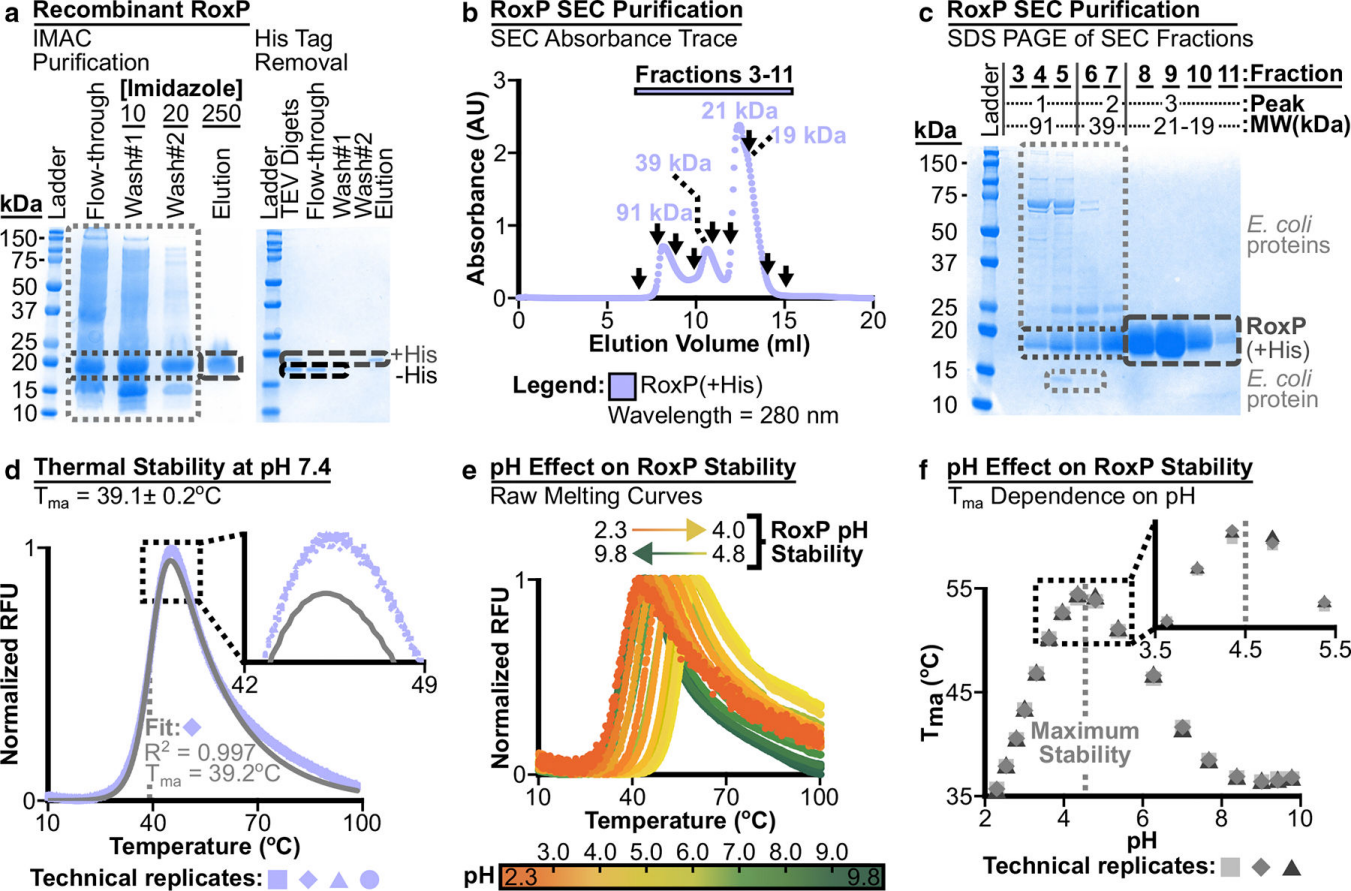
RoxP biochemical evaluation. Recombinant RoxP_1 was (**a**) purified, and then its (**b**, **c**) molecular size/weight, (**d**) thermal stability, (**e, f**) and pH stability were assessed. (**a**) RoxP_1 purified from *E coli* lysate using IMAC was >95% pure, and TEV digest of this protein followed by IMAC capture of His-tagged proteins produced tagless RoxP_1. High MW protein ladder bands (75, 150 kDa) are indicated by a black tick mark. Short-dashed boxes: light gray (E *coli* proteins and lysozyme [15 kDa band]); dark gray (RoxP_1). Long-dashed boxes (>95% pure RoxP_1): dark gray (+His tag); black (−His tag = tagless). IMAC experiment details: left gel (6.0 L culture, 0.5 ml Ni-NTA, gravity flow, single application of lysate, 22.0 mg yield of RoxP_1^a^), right gel (22.0 mg RoxP_1 cut with 12 mg TEV at 4 °C for 72 hours^b^, ~75% tag cleavage, 2.0 ml Ni-NTA, gravity flow, 25.0 mg yield of tagless RoxP_1^c^). Imidazole concentration is in mM. (**b**) SEC was used to evaluate IMAC-purified RoxP_1 (recovery run #2^a^). The major peak/shoulder has predicted MWs consistent with monomeric RoxP_1. Peak 1 is likely >91 kDa, because the fractionation range of this column (Superdex 75 Increase 10/300 GL) is 70–3 kDa. SEC profile evaluated by SDS-PAGE in panel **c** is indicated with individual fractions marked (↓). Absorbance saturation at ~12.5 ml occurred owing to application of more sample (0.6 ml of 100 mg/ml) than normally recommended for this column (eg, ≤50 mg/ml, ≤500 ml). Supplementary Figure S5a provides details for SEC MW calibration curve. (**c**) SDS-PAGE of SEC fractions in **b** indicated with (↓) is shown with boxes as described in **a**. Peaks and their associated MWs thatwere assessed by specific fractions are indicated above the gel. An equal volume of each fraction was loaded per lane. (**d**–**f**) DSF was used to ascertain the apparent melting temperature (T_ma_) of RoxP_1 in (**d**) HSB (20 mM HEPES pH 7.4, 150 mM NaCl, 0.01% azide) and (**e**, **f**) as a function of pH. (**d**, **e**) DSF data are shown as individual data points. (**d**) Data from four replicates and a fit for one replicate are shown. (**e**, **f**) RoxP appears most stable at pH ~4.5 on the basis of both (**e**) raw DSF melting curves and (**f**) T_ma_ values from fitting the raw data. ^a^Two subsequent IMAC purifications (recovery runs #2 and #3) over 6.0 ml of Ni-NTA (3 applications of sample) using this lysate captured 2 82 mg and then 195 mg of RoxP_1 protein, respectively. Thus, the total RoxP_1 protein yield was ~500 mg, and ~83 mg of RoxP_1 protein was produced per liter of culture. ^b^TEV digest was optimized (eg, lower protein mass ratio, shorter incubation time) after this experiment. ^c^Pre-/post-TEV digest RoxP_1 yield discrepancy is most likely due to 10 mM imidazole present in the protein sample used for the TEV digest. As such, the final RoxP_1-tagless yield was ~95 mg per liter of *E coli* culture. DSF, differential scanning fluorimetry; HSB, HEPES sizing buffer; IMAC, immobilized metal affinity chromatography; MW, molecular weight; NaCl, sodium chloride; SEC, size-exclusion chromatography.

Before assessing RoxP ligand binding, we biochemically characterized the ligand-free (apo) form of RoxP. We chose to work with the RoxP_1 ortholog (RefSeq identification: WP_002515361.1), because it was (i) the most commonly observed ortholog (Figure 1c and d), (ii) encoded in the genome of the first sequenced *C acnes* strain (KPA171202) (Bruggemann, 2004), and (iii) previously assessed for heme-binding in the literature (Allhorn et al, 2016; Stødkilde et al, 2022). RoxP_1 was cloned downstream of an eight histidine (denoted as 8His) tag followed by a TEV (Tobacco Etch Virus) cleavage site (Supplementary Material). This construct was then recombinantly expressed in *Escherichia coli;* 8His-TEV-RoxP_1 was purified from *E coli* lysate using immobilized-metal affinity chromatography (IMAC), and tagless RoxP protein was produced through TEV digest followed by IMAC capture of tagged proteins (Figure 3a). Size-exclusion chromatography (SEC) showed that 8His_TEV_RoxP_1 elutes predominantly as a single, monodispersed peak at a SEC-estimated molecular weight (MW) of 21 kDa (Figure 3b and c). This MW is consistent with the monomer MW (i) predicted after PelB signal peptide cleavage (17.2 kDa) and (ii) observed by SDS-PAGE for tagged RoxP (Figure 3a and c) (MW = ~19 kDa, +His), as well as tagless-RoxP (−His) produced after TEV proteolysis (Figure 3a) (MW = 14.9 kDa).

Recombinant RoxP_1 did not appear to be bound to an endogenous *E coli* porphyrin, because absorbance scans from 220 to 800 nm did not identify peaks beyond the 280 nm protein peak. Therefore, RoxP_1 appeared to be purified in its apo-form. Apo-RoxP_1-tagless could then be concentrated to ~282 mg/ml, which suggested that it was a highly stable protein. Surprisingly, differential scanning fluorimetry (DSF) assessment of the apo-RoxP_1-tagless apparent melting temperature (T_ma_) in SEC buffer (HEPES sizing buffer: 20 mM HEPES pH 7.4, 150 mM sodium chloride, 0.01% azide) revealed that it has very low T_ma_ of 39.1 ± 0.2 °C (Figure 3d). Since human skin structures (eg, PSUs) exist across a vertical temperature gradient (deep: 37 °C; surface: 31.6 °C [Benedict et al, 1919]) that also varies regionally across the face (30.4–36.9 °C [Benedict et al, 1919; Bierman, 1936; Lee et al, 2019]), we determined whether RoxP might be more stable under conditions more like those found in the PSUs. We examined the effect of pH on apo-RoxP_1-tagless and found that (i) low pH stabilized this protein’s T_ma_ by >15 °C (Figure 3e and f) and (ii) it appeared to be most stable at pH ~4.5, which is consistent with the pH 4.0–5.5 found on forehead skin (Prakash et al, 2017; Zlotogorski, 1987).

### Assessment of RoxP ligand binding

**Figure 4.**
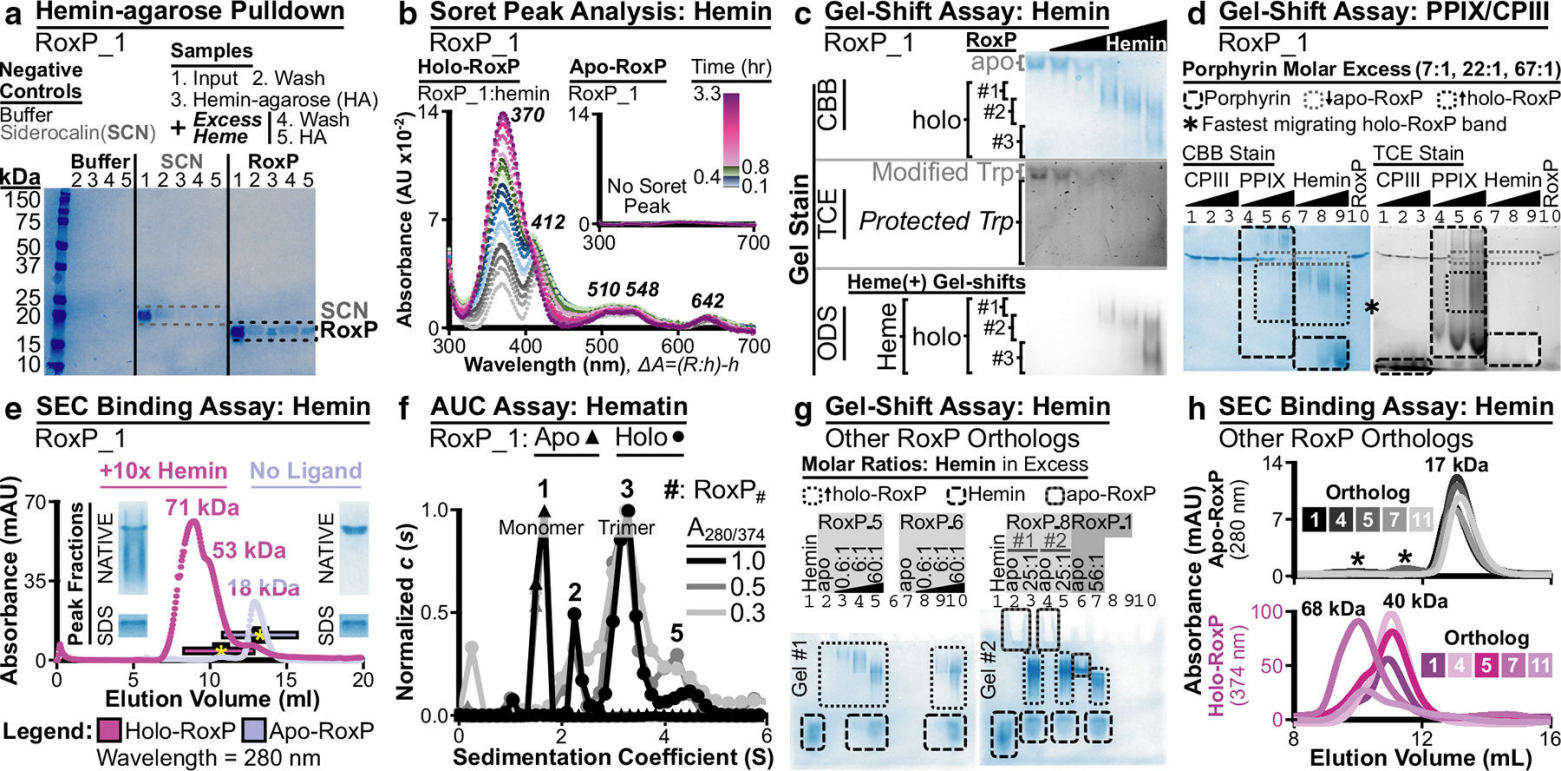
Assessment of RoxP:porphyrin binding. Using tagless, recombinant RoxP proteins, porphyrin-binding assays were performed for (**a**–**h**) RoxP_1 and (**g**, **h**) other RoxP orthologs. (**a**) HA pulled down RoxP_1 (RoxP, lane 3), but it did not significantly pull down a similarly sized, tagless, NCP involved in human nutritional immunity (SCN, lane 3). Preincubation with excess of hemin did not prevent HA pulldown of RoxP_1 (RoxP, lane 5), but it eliminated nonspecific, HA pulldown of NCP (SCN, lane 5). SCN and RoxP bands are outlined by dashed gray and black lines, respectively. (**b**) Soret peak analysis of holo-RoxP (RoxP_1:hemin, 10:1 molar ratio) was compared with an inset of the same analysis performed on apo-RoxP (RoxP_1). Scans were taken over 3.3 hours. Soret peaks and Q-bands are shown in bold italics. Incubation time prior to each scan is shown in the legend (top right) as a color gradient. Color gradient heights were scaled to the time span examined. From bottom to top, the color gradient and number of included traces for each incubation time span are gray scale (0–240 s, 5 traces), blue (300–1600 s, 5 traces), green (1560–2760 s, 3 traces), and magenta (2820–11,819 s, 6 traces). All spectra were blank subtracted. X-axis label shows the difference spectra calculation. Absorbance is denoted by A, RoxP is denoted by R, hemin is denoted by h, and RoxP:hemin is denoted by R:h. (**c**, **d**) NATIVE gel-shift assessment demonstrated RoxP_1 porphyrin binding of (**c**) hemin and (**d**) PPIX but not CPIII. (**c**) RoxP:heme binding was shown by titratable, hemin-dependent (i) decrease in apo-RoxP; (ii) increase in holo-RoxP; (iii) protection of RoxP W66 by heme-binding; and (iv) the comigration of heme with three distinct holo-RoxP species. TCE = W protection assay; ODS = heme stain. Supplementary Figure S3 shows the images of entire gels that include control lanes (eg, hemin alone, NCPs). (**d**) RoxP:PPIX binding was shown by titratable, PPIX-dependent (i) decrease in apo-RoxP and (ii) increase in holo-RoxP visualized by both CBB and TCE. As opposed to hemin and CPIII that migrate to the bottom of the gel (Figure 4d and Supplementary Figure S4d and e), PPIX alone at 375 pM (67:1 molar PPIX concentration) displays distinct CBB and TCE staining patterns that extend throughout the lane but are distinct from holo-RoxP bands (Supplementary Figure S4a and b, lane 2). (**e**) SEC was performed on apo-RoxP_1 alone and preincubated with 10-fold molar excess of hemin. Holo-RoxP eluted significantly earlier than apo-RoxP with a higher 280 nm absorbance (A_280_) and NATIVE gel-shift bands consistent with holo-RoxP. This SEC peak contained NATIVE gel-shift bands (Supplementary Figure S5c–f) that exhibited tryptophan protection (TCE negative) and heme comigration (ODS positive). Comparison of apo-/holo-RoxP peak SDS-PAGE gel samples suggests that the increased holo-RoxP A_280_ (~36 mAU) is due to hemin, because the apo-RoxP peak fraction contained 10-fold more protein than the holo-RoxP peak fraction (Supplementary Figure S5g). Supplementary Figure S5a shows SEC MW calibration curve, and Supplementary Figure S5d and g shows uncropped gel images (NATIVE, SDS-PAGE) shown in this panel. The cropped gel images in panel **e** are from the fractions marked with a yellow asterisk (*), and all fractions examined in Supplementary Figure S5c–g are shown as a black-outlined bar with the same color as the A_280_ trace. (**f**) AUC was performed on apo-RoxP_1 and SEC-purified holo-RoxP_1 (RoxP:hematin) to assess heme-dependent oligomerization. Supplementary Figure S5b shows the MW calibration curve of SEC used to prepare holo-RoxP_1. (**g**) NATIVE gel-shift assessments demonstrated RoxP_5, 6, 8 all bind heme. Gel #1 Apo-RoxP_5/6 (pI 9.56) did not enter the gel run with forward polarity, whereas gel #2 apo-RoxP_8 (pI 8.98) slightly entered the borders of the wells (lanes 2 and 4) and disappeared upon heme-binding (lanes 3 and 5). Apo-RoxP_1 (pI 6.19) entered the gel #2 (lane 6), as seen in **c** and **d**. Single biological replicates are shown for RoxP_5/6, and two biological replicates are shown for RoxP_8. Unlabeled lanes were loaded with blank samples. (**h**) SEC analysis of RoxP_1, 4, 5, 7, 11 demonstrated a heme-dependent increase in MW (+23–51 kDa) for all assessed orthologs. Absorbance at 374 nm (A_374_) was used to monitor heme-bound RoxP (holo-RoxP). Holo-RoxP A_280_ peaks coincided with A_374_ peaks due to both protein and hemin absorbing at 280 nm. Apo-RoxP and hemin alone did not contain any A_374_ SEC trace peaks. Asterisks mark small A_280_ peaks (<1 mAU) that eluted prior to (RoxP_4: 70 kDa; RoxP_1,5: 35 kDa) the main apo-RoxP peak (17 kDa). AUC, analytical ultracentrifugation; CBB, Coomassie brilliant blue; CPIII, coproporphyrin III; HA, hemin-agarose; MW, molecular weight; NCP, negative control protein; ODS, O-dianisidine; PPIX, protoporphyrin IX; SCN, siderocalin; SEC, size-exclusion chromatography; TCE, 2,2,2-trichloroethanol.

After characterizing recombinant apo-RoxP_1-tagless, we assessed RoxP’s ability to bind heme. First, heme-binding was demonstrated for apo-RoxP_1-tagless by hemin-agarose pulldown (Figure 4a). A negative control protein of similar MW (hSCNkk, Supplementary Material) was not significantly pulled down by hemin-agarose. Preincubation with hemin did not prevent RoxP_1 pulldown, which may support hemin exchange during the experiment, as was observed for hemoglobin (data not shown). Soret peak analysis then demonstrated that RoxP:hemin binding reached equilibrium by 2.3 hours in HEPES sizing buffer at room temperature. Soret peak analysis also revealed evidence of both penta- (370 nm) and hexa- (412 nm) coordinated complexes (Rathod et al, 2023) (Figure 4b). In comparison, heme-binding control proteins bovine serum albumin (BSA) and human hemopexin (Hmp) reach equilibrium in this assay at 1.8 and 0.3 hours, respectively (Supplementary Figure S2a–d). The time required for BSA/Hmp:hemin to reach equilibrium has previously been attributed to slow dissociation of hemin dimers into monomers in aqueous solutions (Kuželová et al, 1997). Heme preparation (hemin in DMSO vs hematin in 1.4 N sodium hydroxide) was also found to produce similar RoxP Soret peaks (Figure 4b and Supplementary Figure S2e, g, and h).

The conditions assessed by this spectroscopy work were used to develop a NATIVE gel-shift assay that revealed heme titration–dependent (i) appearance of new, faster migrating bands (holo-RoxP) by Coomassie brilliant blue staining; (ii) protection of RoxP’s partially buried tryptophan (W66) from 2,2,2-trichloroethanol modification (Ladner-Keay et al, 2018); and (iii) heme staining of the new, faster migrating bands (Figure 4c and Supplementary Figure S3). NATIVE gel-shift analysis of two *C acnes* porphyrins (Cornelius and Ludwig, 1967; Lee et al, 1978) revealed weaker binding to PPIX but no detectable binding to coproporphyrin III (CPIII) (Figure 4d) even after extended incubation (0.5 hours to overnight) (Supplementary Figure S4a–e). Similar to hemin, PPIX protected apo-RoxP_1 W66 from 2,2,2-trichloroethanol modification (Figure 4d and Supplementary Figure S4b). Unlike hemin though, PPIX also produced a distinct 2,2,2-trichloroethanol–positive gel-shift coincident with the RoxP_1:PPIX Coomassie brilliant blue gel-shift, likely due to 2,2,2-trichloroethanol modification of metal-free porphyrins (Supplementary Figure S4b [PPIX] and d [CPIII]).

Weak/absent RoxP binding of *C acnes* porphyrins may be due to the assayed condition (HEPES sizing buffer, pH 7.4) being quite different from the low pH, lipid-rich PSU that *C acnes* normally inhabits. This hypothesis is supported by an observation that we made during the development of our RoxP:hemin NATIVE gel-shift assay. A minor increase in running buffer pH from 8.3 to 8.6 decreased RoxP:hemin gel shifts, suggesting a decrease in affinity (Supplementary Figure S4f and g).

Although NATIVE gel-shift band migration is not always an indicator of a change in oligomeric state, two observations from this work suggested this that might occur with RoxP:heme binding. First, the appearance of multiple new bands with hemin titration (Figure 4c and Supplementary Figure S3) suggested that there were multiple RoxP:heme species present. Second, extended incubation (0.5 hours to overnight) favored the appearance of a distinct, faster migrating holo-RoxP species (Supplementary Figure S4e, g, and h). These observations led us to assess the holo-RoxP oligomeric state using SEC and analytical ultracentrifugation (AUC). SEC suggested that heme-binding increased RoxP’s MW (Figure 4e), whereas AUC demonstrated several RoxP:heme oligomeric states (trimer >> dimer > pentamer) (Figure 4f). Heme-binding after SEC was confirmed by NATIVE gel-shift analysis (Figure 4e and Supplementary Figure S5c–f), as well as heme-specific absorbance at 374 nm (Figure 4f). These findings revealed that RoxP_1 undergoes heme-dependent oligomerization (HDO), which had not been previously identified.

### Evolutionary conservation of RoxP biochemistry and ligand binding

Owing to RoxP’s high sequence conservation (Figure 1), we next sought to determine whether heme-binding or oligomerization is conserved across RoxP orthologs. Three RoxP orthologs (RoxP_5, 6, 8) with 81–82% sequence identity to RoxP_1 were initially shown (Figure 4g) to bind hemin through NATIVE gel-shift assays. RoxP_8 also demonstrated heme-binding by Soret peak analysis (Supplementary Figure S2f). These findings led us to extend our SEC heme-binding assessment to include three additional RoxP orthologs (RoxP_4, 7, 11), which demonstrated that heme-binding is conserved across RoxP orthologs with sequence identity from 71 to 100% (Figure 4h). Furthermore, the increased MW of the holo-RoxP_4/5/7/11 complexes suggest that HDO is also conserved (Figure 4h). Owing to the conservation of heme-binding and HDO across seven RoxP orthologs (Supplementary Table S5), we extended our DSF pH stability analysis and found that low pH stabilization was also conserved (Supplementary Figure S6) for RoxP_5 (82% identity) and RoxP_11 (71% identity), because both were most stable at pH 4.8 and 4.5, respectively. These results suggested that RoxP function at low pH is highly conserved across RoxP orthologs.

### Molecular modeling to identify RoxP HBS

**Figure 5.**
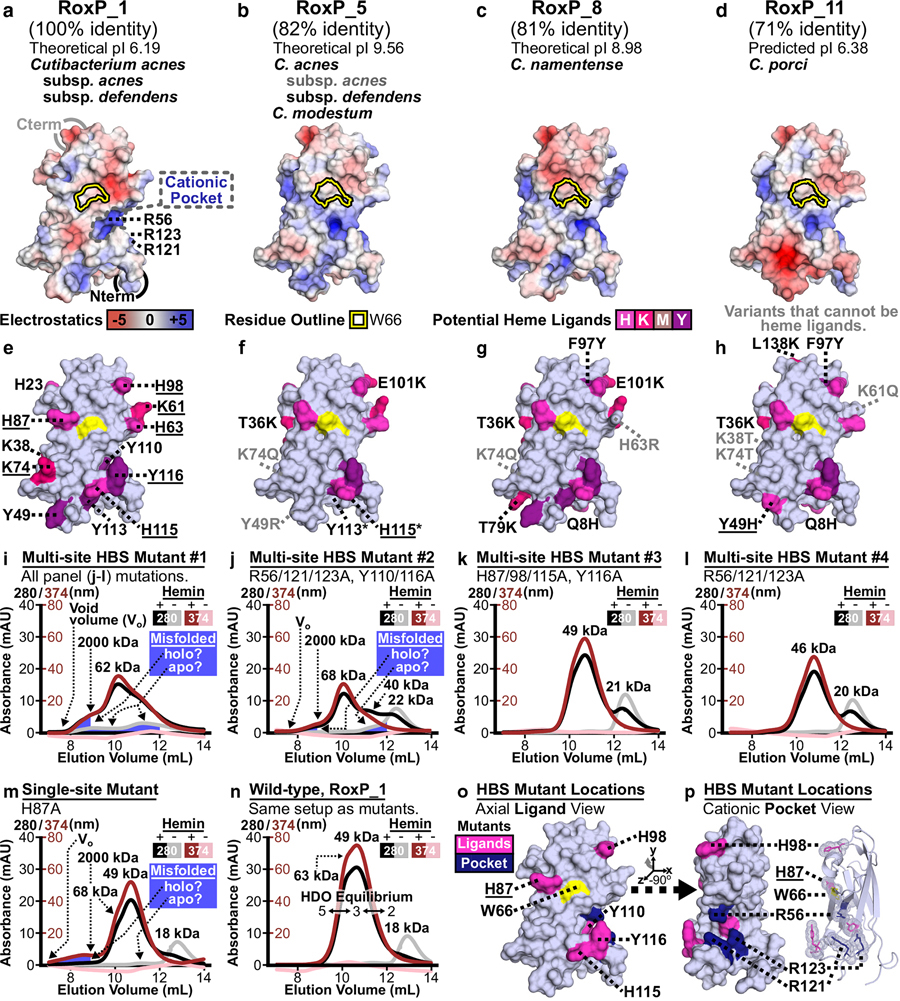
Identification of a RoxP HBS. Molecular modeling was used to identify potential RoxP HBS residues, and site-directed alanine mutagenesis was used to evaluate the contribution of these residues to HDO. (**a**–**d**) For each heme-binding RoxP ortholog (Supplementary Table S5), sequence identity relative to RoxP_1, theoretical pI, and *Cutibacterium* species/subspecies that encode the ortholog are shown. (**a**–**d**) Electrostatic surfaces and (**e**–**h**) potential heme ligands are shown for (**a, e**) the NMR structure of RoxP_1 (PDB 7bcj) and (**b**–**d**, **f**–**h**) three-dimensional models of three RoxP orthologs (RoxP_5, 8, 11). The partially buried tryptophan surface is (**a**–**d**) outlined or (**e**–**h**) colored yellow (Figure 2) on each molecular surface, and the (**a**) cationic pocket is outlined by a gray, dashed line. (**b**) The text “subsp. *acnes”* is labeled gray, because the single IPG entry ofa *C acnes* subspecies *acnes* RoxP_5 may be due to a misannotation of that IPG entry. (**e**–**h**) Potential RoxP heme axial ligands are labeled in black, underlined if in close proximity to W66, and colored according to Figure 2**d**. (**f**–**h**) Ortholog positions lacking a potential heme axial ligand found in RoxP_1 are labeled in gray. (**e**) Three potential RoxP_1 heme axial ligands (Y105, K130, and M133) located on the molecular face opposite that containing the partially exposed tryptophan residue were not labeled. (**f**) Asterisks mark side chains that flipped during modeling of RoxP_4, 5, 6, 11, which might make it difficult to identify two potential, invariant heme ligands (Y113 and H115). In the RoxP_7/8 models, only the H115 side chain flipped. Supplementary Figure S7 shows this analysis of three additional heme-binding orthologs (RoxP_4, 6, 7). (**i**–**n**)

The high degree of RoxP functional conservation led us to assess what conserved structural determinants might support these functions. Phyre2 (Kelley et al, 2015) was used to generate three-dimensional models of six RoxP orthologs (RoxP_4–8, 11) that bind heme (Supplementary Table S5). Electrostatic analysis in PyMOL (version 2.5.2) (Schrödinger, 2022) of these models (Figure 5a–d and Supplementary Figures S7a–d and S8) identified a cationic pocket immediately below the invariant tryptophan (RoxP_1 W66). This pocket contains three arginines (R56, 121, 123) and two tyrosines (Y110, 116) that are invariant across all 21 RoxP orthologs. The positive charge in this pocket could provide an ideal environment to bind one of heme’s two negatively charged propionic acid side chains at positions C(2) and C(18) of the porphyrin ring (Sulkowski et al, 2016). Unlike this area of charge conservation, there were also differences in charged surfaces leading to a range of predicted isoelectric points (pl 6.19–9.56), although these differences were primarily on the RoxP molecular surface opposite that of the conserved HBS (Supplementary Figure S8).

Next, we sought to identify RoxP’s heme axial ligand(s). Five different amino acids (C, H, K, M, and Y) can serve as axial ligands to heme iron, although histidine is the dominant residue (~80%) in both heme b and heme c types (Li et al, 2011). Several potential heme axial ligands were easily eliminated from consideration on the basis of their (i) sequestration within a disulphide bond (C43-C111) or (ii) location (Supplementary Figure S1f and Figure 5e) on the molecular face distant from W66 (H23, K38, Y105, K130, M133). Furthermore, several ortholog variants that introduced new potential heme ligands (Figure 5f–h and Supplementary Figure S7f–h) could be eliminated, because they were (i) not found in RoxP_1 or (ii) >25 Å from W66. Then, an assessment of conservation of potential ligands across heme-binding orthologs (Figure 5e–h and Supplementary Figure S7e–h) and the distance of these residues to W66 (Supplementary Table S6) was able to reduce eight potential ligands to four probable ligands (H87 > Y116, H98 > H115). These residues encircle W66 at clock positions 1(H98), 5(H115, Y116), and 10(H87)-o’clock. Since the heme iron’s location is unknown, any of these four residues could serve as heme axial ligands.

Site-directed alanine mutagenesis followed by SEC HDO analysis (Figure 4h) was used to assess the contribution of these four residues and other potential HBS contacts. HBS mutant #1 SEC suggested that its mutations impede native protein folding (Figure 5i), whereas the appearance of small shoulders of misfolded/aggregated protein in two other panels (Figure 5j and m) was consistent with small decreases in protein stability (Supplementary Material). All interpretable multisite mutants (Figure 5j–l) also showed a small increase in the apparent MW of apo-form, which may be due to their mutations increasing loop 112–123 flexibility, leading to a larger hydrodynamic radius in solution.

The SEC profiles of HBS mutant #2 (Figure 5j) were interpretable and suggested that at least one mutated residue impaired HDO. Restriction of alanine mutation to the four probable heme axial ligands (Figure 5k) or cationic pocket arginines (Figure 5l) also resulted in interpretable SEC profiles, which demonstrated that mutating these residues decreased HDO. Part of the HDO decrease observed in Figure 5k could be replicated by mutating H87A (Figure 5m), which suggests that this is one of the four Figure 5k mutations that decrease HDO.

In comparison with wild-type RoxP_1 run under the same experimental conditions (Figure 5n), four mutants clearly showed an increase in apo-form (A_280_ peak without a comigrating A_374_ peak) when heme was present (Figure 5j–m). This result helped us to refine our RoxP HBS footprint (Figure 5o and p) and supports a role for both interfaces in HDO. This analysis suggested that RoxP function requires two highly conserved molecular surfaces that could be targeted diagnostically to better (i) study *C acnes* adaptation to human skin and (ii) identify *C acnes* infections of humans.

### Development of anti-RoxP immunoassays

#### Indirect ELISA.

**Figure 6.**
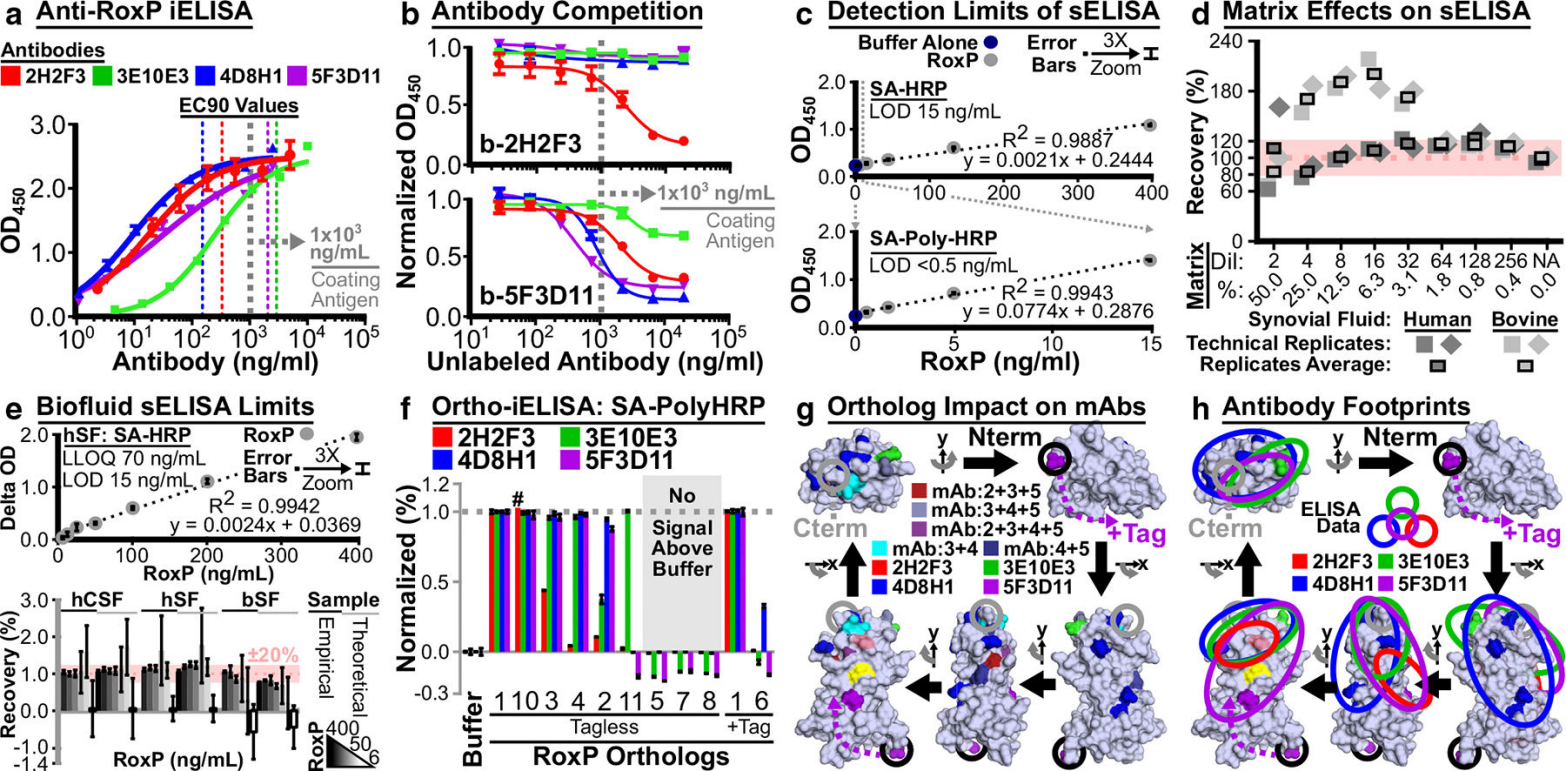
Anti-RoxP immunoassay development. (**a**) iELISA curves showing binding of recombinant apo-RoxP_1-tagless by antibodies 2H2F3, 3E10E3, 4D8H1, and 5F3D11. Two-layer detection: (i) biotinylated, goat antimurine IgG and (ii) SA-HRP. (**b**) ciELISA curves showing RoxP detection by2H2F3, 3E10E3, 4D8H1, and 5F3D11 antibodies (see **a** for mAb colors) in the presence of (top panel) biotinylated 2H2F3 (b-2H2F3) and (bottom panel) b-5F3D11. (**c**) Linear range of anti-RoxP sELISA using 4D8H1 (capture mAb), b-2H2F3 (detection mAb), and two versions of SA-HRP: SA-HRP and SA-Poly-HRP. All samples were run with three technical replicates. (**d**, **e**) Horizontal dotted red line: 100% recovery. Pink box: ±20% recovery. (**d**) The sar-sELISA approach was used to determine the matrix effect of synovial fluid on the anti-RoxP sELISA. Matrix in each sample is shown as dilution (denoted as Dil) and volume-to-volume (v/v) percentage (%). (**e**) After sample dilution (8-fold: hSF/CSF, 4-fold: bSF), a 2-fold RoxP dilution series (400–6.25 ng/ml) was used to determine the linearity (top: hSF) and limit of detection (bottom: hCSF, hSF, bSF) for the anti-RoxP sELISA in complex samples. (**f**) Ten recombinant RoxP orthologs were evaluated by iELISA (coating concentration: 10 mg/ml; terminal detection reagent: SA-Poly-HRP) in technical triplicate for binding by2H2F3, 3E10E3, 4D8H1, and 5F3D11. Supplementary Material provides explanation of negative signal. ^#^Marks a single RoxP_10–2H2F3 replicate due to two miss-seated micropipettor tips, but 100% 2H2F3 binding was observed for this ortholog under all other conditions (Supplementary Figure S15). (**g**) On the basis of Supplementary Table S7, RoxP ortholog variant positions that impact antibody binding were mapped to PDB 7bcj and colored according to which antibodies were impacted by a variant. For example, cyan residues (28–30) only affected 3E10E3 and 4D8H1 (3 + 4), whereas the violet–purple residue 89 affected all mAbs (2 + 3 + 4 + 5).(**h**) Antibody footprints were mapped onto RoxP_1 using variant positions (i) unique to each antibody (**g**, **h**) and (ii) all variants impacting an antibody (Supplementary Figure S16). (**g**, **h**) Model x/y-axis rotations, RoxP_1/W66 surface coloring, and N-/C-termini circles are the same as in Figure 2. RoxP P91 is not surface exposed, so its location and effect on 2H2F3 binding are indicated bya red oval with 30% transparency in the bottom-left model. The proposed extension of the N-terminal tag causing partial inhibition of 5F3D11 (**f** and Supplementary Figure S15) is shown as “±Tag.” (**a**–**c**, **e**, **f**) Error bars represent SD. bSF, bovine synovial fluid; ciELISA; competition indirect ELISA; CSF, cerebrospinal fluid; hCSF, human cerebrospinal fluid; hSF, human synovial fluid; iELISA, indirect ELISA; OD, optical density; SA-HRP, streptavidin-horseradish peroxidase; SA-Poly-HRP, streptavidin-poly-horseradish peroxidase; standard deviation, SD; sELISA, sandwich ELISA; sar-sELISA, spike-and-recovery sandwich ELISA. Comparison of SEC HDO analysis of (**i–m**) RoxP_1 HBS mutants to (**n**) wild-type RoxP_1 demonstrated that (**j–m**) four mutants decreased HDO. All experiments were performed on the same SEC 70 column using the same conditions as that described for Figure 4h. SEC data point color indicates the absorbance wavelength and presence/absence of hemin. Peak/shoulder calculated MWs (Supplementary Figure S5b) are shown. (**i, j, m**) The SEC 70 column void volume (V_o_) and the elution volume of blue dextran (~2000 kDa) were labeled in these panels, because these mutants have absorbance profiles in these regions consistent with misfolded, aggregated protein, which suggest that the ~40 kDa apo-form shoulder is misfolded protein and not a specific RoxP oligomeric state. (**i**) Since HBS mutant #1 did not have a monodispersed apo-peak, it did not appear to be properly folded, so the effect of its mutations on HDO could not be assessed. (**n**) Under these experimental conditions, wild-type RoxP_1 almost completely converts apo-form to holo-form with calculated MWs consistent with the AUC-determined HDO equilibrium (pentamer ⟷ trimer ⟷ dimer). (**o, p**) Evaluated alanine mutants are shown in relation to W66 as (**o**) axial ligand and (**p**) cationic pocket views, with residue positions colored according to proposed function. H87 was underlined to emphasize its confirmation as contributing to the HBS in **m**. (**e, o**) Same view of the apo-RoxP_1 NMR surface. (**p**) A y-axis rotation of the (**o**) surface shown next to a cartoon representation, with mutations shown as sticks/dots to highlight the proximity of loop mutants that may explain how alanine mutagenesis in the loop region increases the apparent apo-form MW. AUC, analytical ultracentrifugation; HDO, heme-dependent oligomerization; HBS, heme-binding site; IPG, Identical Protein Group MW, molecular weight; NMR, nuclear magnetic resonance; SEC, size-exclusion chromatography.

To further investigate RoxP function, we generated four murine, IgG, anti-RoxP antibodies (2H2F3, 3E10E3, 4D8H1, and 5F3D11) against RoxP_1-tagless and then validated their specificity using an anti-RoxP indirect ELISA (iELISA) (Figure 6a). This assay revealed that three antibodies had a higher affinity for RoxP_1 (4D8H1 > 2H2F3 > 5F3D11 >> 3E10E3). To simplify anti-RoxP immunoassay development, these antibodies were biotinylated and then validated by iELISA (Supplementary Figure S9), which revealed that biotinylated 3E10E3 is not able to detect RoxP_1. Sequencing of the fragment variable regions of these antibodies (Supplementary Material) suggested that biotinylated 3E10E3 was biotinylated at a complementarity determining region lysine required for antigen binding.

#### Competition iELISA.

Biotinylated antibodies (b-2H2F3, b-4D8H1, b-5F3D11) were then used in competition iELISAs against a titration of all four unlabeled antibodies (Figure 6b and Supplementary Figure S10). Although multiple antibodies could compete against b-5F3D11, b-2H2F3 and b-4D8H1 were only out competed by their unlabeled counterparts. The 5F3D11 competition data suggested that the binding site of this antibody sterically overlaps with those of the other three antibodies, whereas the lack of competition between 2H2F3 and 4D8H1 suggested that these antibodies bind nonoverlapping epitopes.

#### Sandwich ELISA.

These noncompetitive antibodies (4D8H1, 2H2F3) were then evaluated in both orientations to develop an anti-RoxP sandwich ELISA (sELISA). The best orientation (capture: 4D8H1;detection: biotinylated 2H2F3) was shown to be linear and to have a limit of detection that could be enhanced by >30-fold from 15 to <0.5 ng/ml RoxP_1 (Figure 6c). To determine whether this sELISA could be used to identify *C acnes* infections, we performed spike-and-recovery sELISA in human synovial fluid, CSF, and serum;bovine synovial fluid;and *C acnes* growth media (Figure 6d and Supplementary Figure S11). Unlike human synovial fluid (hSF), the bovine synovial fluid matrix effect increased between spike-and-recovery sELISA experiments (#1 [Supplementary Figure S11d] and #2 [Figure 6d]), which suggests that the intervening biofluid storage at −20 °C affected bovine synovial fluid but not hSF. Matrix effects identified by spike-and-recovery sELISA demonstrated that all complex fluids required dilution (1/4 or 1/8) to recover 100 ± 20% of the spiked in RoxP_1 with stable recovery upon further dilution.

Using this dilution information, sELISA linearity of dilution, limit of detection, and lower limit of quantitation were assessed in all six complex fluids (Figure 6e and Supplementary Figure S12). Using the streptavidin-horseradish peroxidase sELISA approach, the assessed limit of detection ~15 ng/ml and lower limit of quantitation ~70 ng/ml were consistent with the percentage recovery limit of 50–100 ng/ml, which would be expected to be enhanced >30-fold to 2–3 ng/ml with the use of streptavidin-poly-horseradish peroxidase. The anti-RoxP sELISA was then used to assess endogenous RoxP secretion from mid-log RCM cultures (n = 5) of two *C acnes* reference strains (KPA171202: 65.52 + 26.09 ng/ml, ATCC 6919: 23.58 + 6.67 ng/ml). Prior literature noted that stationary-phase culture of KPA171202 contained ~10 μg/ml RoxP (Allhorn et al, 2016), which suggests that RoxP accumulates during culture and may be expressed at higher levels during stationary phase. As such, this sELISA appears to be able to assess RoxP secretion both in vitro and in vivo.

#### Ortholog specificity of anti-RoxP immunoassays.

Although RoxP is highly conserved, our analysis of nonconservative amino acid solvent exposure on RoxP (Figure 2b and Supplementary Figure S1c) suggested that sequence variation might inhibit anti-RoxP antibody recognition of some clinically relevant RoxP orthologs (eg, Supplementary Tables S2 and S3: RoxP_2 – Asn12, RoxP_5 – ATCC 11828). Ten RoxP orthologs (RoxP_2–11) that represented a wide range of variation compared with RoxP_1 (71–99% identity, 1–39 amino acid differences) (Supplementary Figure S13) were selected to determine the ortholog specificity of our anti-RoxP antibodies. Tagless, recombinant RoxP was prepared using a small-scale protocol (Supplementary Figure S14) and then assessed by anti-RoxP iELISA (Figure 6f and Supplementary Figure S15). Although RoxP orthologs with >90% identity to RoxP_1 were bound by many antibodies, even a small number of variant positions could impede antibody binding. For example, RoxP_2 has three variant positions, which result in significant inhibition of both 2H2F3 and 3E10E3 binding. With greater sequence divergence from RoxP_1, the success rate of ortholog detection by anti-RoxP antibodies decreased, but not all antibodies were impeded by significant differences in ortholog sequence identity to RoxP_1 (eg, RoxP_6, 81%; RoxP_11, 71%).

#### Anti-RoxP antibody footprinting analysis.

Variable ortholog recognition gave us the opportunity to perform antibody footprinting to localize anti-RoxP epitopes. Antibody binders were compared with partial/nonbinders to identify variants near/within antibody footprints (Figure 6f and Supplementary Figure S15 and Supplementary Material). Those ortholog variant positions (Supplementary Table S7) were mapped to the apo-RoxP_1 NMR structure (PBD 7bcj) (Figure 6g and Supplementary Figure S16) and used to identify potential antibody binding footprints (Figure 6h and Supplementary Material). These antibody footprints largely reproduced our competition iELISA findings (Figure 6b and Supplementary Figure S10) and are supported by complementarity determining region conservation between antibodies (Supplementary Material). Interestingly, all four antibodies were affected by G89, which effectively pinned their footprints to a single location near the C-terminus (Figure 6g). Although three of these antibodies have footprints that appear to overlap the C-terminus (3E10E3/4D8H1 > 5F3D11), the 5F3D11 footprint appears to extend away from the C-terminus toward the antioxidant tyrosine loop. The antibody footprint locations relative to W66 and the conserved cationic pocket also suggest that heme-binding may affect 5F3D11 > 2H2F3 > 3E10E3 > 4D8H1 (Figure 6h and Supplementary Figure S16).

Because an antibody typically buries ~500 Å^2^ of antigen solvent-accessible surface area (Reis et al, 2022; Rubinstein et al, 2008), it is quite remarkable that these four antibodies have distinct footprints on a protein as small as RoxP (solvent-accessible surface area of 8,836.82 Å^2^). Even more striking is the fact that two of these antibodies could be used to develop an anti-RoxP sELISA. Furthermore, the arrangement of these four antibodies on RoxP now provide a unique opportunity for future in vitro studies of RoxP function, as well as the development of *Cutibacterium-specific* clinical diagnostics and antibody-based therapeutics.

## DISCUSSION

RoxP appears to be a *Cutibacteria* adaptation to human skin that arose after they had immigrated from cows to humans but before *C acnes* emerged to dominate human sebaceous skin (Allhorn et al, 2016; Scholz and Kilian, 201 6). As such, RoxP may have played a key role in establishing this microbiome and helped guide host–microbe coevolution during the transition of humans from hunter-gather to agrarian communities. Since no other organism encodes a RoxP homolog, our group decided to perform an in-depth analysis of RoxP sequence space, biochemistry, and function. We then developed immunoassays to measure RoxP in culture media and mammalian biofluids, so that we can investigate this protein’s role in *C acnes* biology and aid in the identification of *C acnes* infections. Through this work, we identified high conservation of RoxP solvent-exposed surfaces, ligand-dependent oligomerization, and low pH stability. This analysis has begun to shed light on RoxP’s function and helps us to understand the unique needs of a PSU microbe. Notably, RoxP low pH stability also suggests how this protein may have helped guide host–microbe coevolution.

### Life on humans favors RoxP conservation

*Cutibacteria* evolution (Scholz and Kilian, 2016) appears to have preceded from older species that do not encode *roxP (C granulosum/avidum)* to intermediary ancestors with high pI RoxP (C *modestum/namnetense* [Dekio et al, 2021a, 2021b; Scholz and Kilian, 2016]). They then evolved into *C acnes* and split into subspecies with high pI RoxP (*C acnes* subspecies *defendens)* or low pI RoxP (*C acnes* subspecies *acnes/elongatum). C acnes* significantly contributes to the low pH of human skin (Marples et al, 1971), and the dominant RoxP ortholog on human skin has a much lower pI (RoxP_1, pI 6.19) than many other orthologs. On the basis of our analysis of RoxP orthologs (Figure 1c and Supplementary Table S2), 93% of *C acnes* subspecies *acnes* RoxP orthologs (n = 90) have this pI, whereas 76% of *C acnes* subspecies *defendens* (n = 55) have a pI > 8.7. *C acnes* subspecies *elongatum* has an even lower pI of 5.90, although it is not associated with acne and seems to preferentially inhabit the less acne-prone skin of the lower back (Barnard et al, 2016a; Dekio et al, 2019; Petersen et al, 2017). Since *C acnes* subspecies *acnes* appears to dominate the majority of human sebaceous skin, it appears that evolution of low pI RoxP may have aided in the emergence of this subspecies.

Strikingly, a comparison of *C acnes* phylotypes between acne and healthy skin (Dagnelie et al, 2018) suggests that decreasing the burden of *C acnes* that encodes low pI RoxP is correlated with healthy skin. As such, we propose that the low pH environment created by *C acnes* supported the evolution low pI RoxP, which helped a pathobiont subspecies *(C acnes* subspecies *acnes)* emerge and then contribute to the human disease acne vulgaris. Interestingly, the only known acne vulgaris cure (isotretinoin) raises skin pH by >0.5 pH (Kmieć et al, 2013), which may support the expansion of healthy *C acnes* (phylotype II, *C acnes* subspecies *defendens* [Dagnelie et al, 2018]) that encode high pI RoxP. In support of this hypothesis, 67% of *C acnes* subspecies *defendens* encode RoxP_5, which is more stable at neutral pH (T_ma_ +8.1 °C) than RoxP_1 (Figure 3d-f and Supplementary Figure S6).

Although it has been established that *C acnes* requires RoxP to colonize human skin (Allhorn et al, 2016), it may have also contributed to *C acnes* dominance of sebaceous skin. *C acnes* constitutes ~50% of skin surface microbes (Grice et al, 2009; McCoy et al, 2019) and ~90% of PSU microbes (Barnard et al, 2016b; Fitz-Gibbon et al, 2013). The emergence of *C acnes* and its rise to dominance likely led to changes in human gene expression, and these changes may have even supported *C acnes* survival in the PSU. Thus, this unique microbial tool may have given *C acnes* a survival advantage that helped it secure its place as the dominant member of the sebaceous skin microbiome.

### RoxP conservation is a *C acnes* Achilles’ heel

RoxP sequence space (946 entries) is dominated by RoxP_1 (67% of entries) and a limited number of other orthologs with similar sequence identity (Figure 1). In fact, 80% of RoxP (RoxP_1, 16, 17, 20) are >99% identical. Furthermore, all *C acnes* phylotypes have been observed to secrete RoxP (Supplementary Table S4), which suggests that this protein is a *Cutibacterium-specific* growth biomarker that can be targeted diagnostically and therapeutically. For such a strategy to work, it would be ideal for anti-RoxP reagents to bind a diversity of RoxP orthologs.

Based on Figure 6f, five RoxP orthologs (RoxP_1/10 > 3 > 2 > 4, 90–100% identity) should be detected by our sELISA antibody pair 2H2F3/4D8H1. The sELISA detection limits for each ortholog will likely vary on the basis of their 2H2F3 affinity, so optimization may be necessary for low-affinity orthologs (eg, RoxP_2–4). RoxP_17 and RoxP_20 are also likely to be recognized by the sELISA owing to the antibody-binding permissibility of their variants G89E and T34I (Supplementary Table S7), respectively. On the other hand, RoxP_16’s single variant position S20F likely precludes sELISA recognition, because it appears that RoxP_11 is not recognized by 4D8H1 owing at least in part to S20I.

RoxP orthologs with low 2H2F3/4D8H1 affinity (RoxP_2–4, 6; ~1% of RoxP entries) could also be readily assessed by converting the sELISA into a competition sELISA where the standard curve would be a fixed concentration of RoxP_1 titrated against recombinant RoxP_2–4, 6. For this reason, we conclude that some version of our sELISA should be able to detect 69% of all RoxP and 72% of *C acnes*–specific RoxP IPG sequences (RoxP_1–4, 6, 10). Furthermore, the reported sELISA should detect 67% of all RoxP, 71% of *C acnes*–specific RoxP, and 93% of *C acnes* subspecies *acnes*–specific RoxP IPG sequences (RoxP_1/10).

The development of this type of assay is critically needed, because *C acnes* is an emerging pathogen that causes the majority of shoulder PJIs, as well as many other IMD infections. Yet, *C acnes* infections are frequently not identified owing to an inability of current diagnostics to quickly and clearly differentiate a “true” *C acnes* infection from *C acnes* contaminants. The latter concern arises from *C acnes* ubiquitous nature, as a false-positive culture result might arise from a patient’s own skin, the skin of a close contact (eg, treating surgeon), or the environment. Recently, two groups have analyzed the *C acnes* genomes from PJIs (shoulder, elbow, hip, knee; 17 patients; 27 isolates) (Ponraj et al, 2022; Salar-Vidal et al, 2021), and our analysis of *roxP* in their genomes (Supplementary Table S2) revealed that 80–92% of *C acnes* PJIs include a strain that encodes RoxP_1. Of note, two closely related orthologs (RoxP_5/21, 99% identical, ~82% identity to RoxP_1) that are not recognized by our antibodies were also present in 40% of “infection-likely” *C acnes* PJIs (5 patients, 15 isolates) (Ponraj et al, 2022). Regardless, this genomic analysis indicates that the ability of our anti-RoxP sELISA to identify *C acnes* IMD infections is likely higher than that predicted by our evaluation of the total RoxP sequence space.

### Other *C acnes* antibodies are not amendable to clinical care

Though *C acnes* antibodies have been previously described (Supplementary Table S8) (McDowell et al, 2011, 2005; Negi et al, 2012; Tunney et al, 1999; Valanne et al, 2005), there is no *C acnes* immunoassay used in routine clinical practice owing to several limitations. For example, *C acnes* antibody PAB is commercially available, but it recognizes a cell-surface antigen that has limited its use to immunohistochemistry (Negi et al, 2012). Furthermore, PAB has only been used to stain acid-fast bodies in sarcoid lymph nodes, and no group has reported using it to identify infection. Another *C acnes* antibody (QUBPa4) (Valanne et al, 2005) binds the secreted protein CAMP (Christie–Atkins–Munch–Petersen) factor 1 (Mayslich et al, 2022; Nakatsuji et al, 2011; Valanne et al, 2005), but CAMP factor 1 does not appear to be secreted by all *C acnes* strains (Mayslich et al, 2022). There is also significant interstrain variation in the expression of *C acnes* CAMP factors 1–5 (Valanne et al, 2005). In comparison, RoxP secretion has been observed to occur in all *C acnes* phylotypes (Holland et al, 2010) and all assessed *C acnes* strains (Supplementary Table S4).

Recently, a Luminex assay using anti–*C acnes* polyclonal antibody (pAb) was used to assess 94 *C acnes* culture–positive hSF samples (Toler et al, 2024). *C acnes* antigen was detected (signal/cut off > 1) in the majority of these samples (65%). Furthermore, this assay only detected *C acnes* antigen in a small percentage of the two negative-control groups: (i) infected by other bacteria (5.8%, n = 103) and (ii) low probability of infection (0.28%, n = 1050). Interestingly, the immunogen for this pAb was *C acnes* strain ATCC 11827, which encodes RoxP_1 (Supplementary Table S2). Unfortunately, the nature of this immunization (eg, intact cells, supernatants, lysate) was not reported, so it is unclear whether one of the pAb targets is RoxP_1 or any other potential *C acnes* growth biomarker. As such, it is not known whether this pAb assay can identify an active *C acnes* infection.

Another group has generated an anti-RoxP pAb and used it to develop a competition ELISA with a limit of detection >80 ng/ml in PBS. This assay can detect RoxP_1 recombinantly produced by *Pichia pastoris* (Andersson et al, 2019) and RoxP_1 endogenously secreted by *C acnes* strain KPA171202 (Ertürk Bergdahl et al, 2019). Owing to the nature of a pAb response, this assay may be able to detect a range of RoxP orthologs, although the authors did not evaluate any other RoxP orthologs. Furthermore, pAb assays can suffer from batch-to-batch pAb variability, which can impede their use. In comparison with this pAb anti-RoxP assay, the monoclonal anti-RoxP sELISA described in this article (i) has >150-fold higher sensitivity, (ii) can detect RoxP in multiple human biofluids, and (iii) has been evaluated against a diverse array of 10 RoxP orthologs (71–100% identity). As such, our assay appears to be more suitable as a clinical diagnostic where the RoxP concentration and ortholog are unknown.

### Other RoxP assays are not amendable to clinical care

RoxP detection has also been reported using capacitive (Ertürk et al, 2018) and surface plasmon resonance (Ertürk Bergdahl et al, 2019) biosensors that were created using a molecular surface imprinting technology. Though the surface plasmon resonance assay is quite sensitive (3.68 ng/ml), these approaches are not yet clinically feasible owing to methodological limitations (eg, severe mass transport in complex samples) and the absence of biosensor equipment in clinical laboratories. In comparison, ELISAs are used in clinical laboratories worldwide, which makes our sELISA easily implementable in established clinical workflows. Furthermore, our sELISA (i) is >7-fold more sensitive than the RoxP surface plasmon resonance assay and (ii) could be converted to a lateral flow immunoassay to create a point-of-care assay for *C acnes* infection detection.

### Future impact of RoxP immunoassays

This manuscript’s immunoassays target a protein secreted only by *Cutibacteria,* which provides us the unique opportunity to improve health care and our understanding of microbe evolution. Use of these assays in clinics would help to address widespread confirmation bias where *C acnes* cultures are discounted owing to the (i) growth of a “more likely” infectious organism (eg, *Staphylococci)* or (ii) assumption that *C acnes* must be a contaminant. Furthermore, identification of *C acnes* infections using these assays would lead to appropriate treatment far sooner than awaiting the slow growth of a *C acnes* culture (5–14 days) (Ellsworth et al, 2020). Notably, such treatment might include targeting both *C acnes* and *Staphylococci,* because these microbes can live in close proximity to one another in the PSU (Jahns et al, 2012) and have been cocultured from IMD infections (Frangiamore et al, 2017; Goldenberger et al, 2021; Sandström et al, 2021; Tunney et al, 1999, 1998).

Beyond the potential impact of these assays on health care, their use in future studies will help us understand RoxP’s role in *C acnes* (i) biology and (ii) interactions with other microbes. Such work will expand our understanding of host–microbe coevolution and thus characterize the ecological shift(s) that occurred in a human microbiome upon *C acnes* arrival to the PSU. To support this future work, our group is already developing reagents that recognize RoxP orthologs that escape detection by our current assays, so that growth by *C acnes* subspecies *defendens/elongatum, C modestum,* and *C namnetense* can be assessed both in vitro and in humans. In summary, future work utilizing this paper’s anti-RoxP immunoassays will shed light on the burden of *C acnes* infections, what role RoxP plays in human disease, and how *C acnes* arose to dominate human sebaceous skin microbiomes.

In conclusion, this study has begun to dissect the molecular determinants of the protein RoxP. This protein helped dairy *Propionibacteria* move from simply adapting to live on human skin (*C avidum/granulosum)* to occupying 25% of the skin and dominating the PSU. Reagents developed as a part of this study have created a highly sensitive sELISA that should be able to detect ~90% of *C acnes* infections of human biofluids. This initial RoxP work has led to a better understanding of commensal microbe adaptation, while providing tools to investigate pathobiont evolution and identify human *C acnes* infections.

## MATERIALS AND METHODS

Previously described protocols were adapted to express recombinant proteins using autoinduction (Studier, 2005); prepare previously described recombinant proteins (6His-TEV [Tropea et al, 2009] and hSCNkk [Shields-Cutler et al, 2016]); assess protein sequence conservation (Edgar, 2004; Landau et al, 2005; Madden, 2002; Okonechnikov et al, 2012); use protein sequences to predict protein biochemical characteristics (Gasteiger, 2003; Petersen et al, 2011; Teufel et al, 2022); analyze and predict protein structures (Fraczkiewicz and Braun, 1998; Jurrus et al, 2018; Landau et al, 2005; Porollo et al, 2004; Schrödinger, 2022; Shindyalov and Bourne, 1998); and perform biophysical experiments (AUC (Laue et al, 1992; Philo, 2023; Schuck, 2000) and DSF (Niesen et al, 2007; Wu et al, 2024, 2023)). Supplementary Material provides further details on these experiments.

## Supplementary Material

SUPPLEMENTARY MATERIAL

Supplementary material is linked to the online version of the paper at www.jidonline.org, and at https://doi.org/10.1016/j.jid.2025.03.048.

## Data Availability

The authors confirm that the data supporting the findings of this study are available within the article or its supplementary materials, which are stored as the following Mendeley Data dataset: McCoy IV, William (2025), “JID-2024–0863.R1 _Supplementary_Materials”, Mendeley Data, V1, https://doi.org/10.17632/f9gych5t5k.1 (https://data.mendeley.com/datasets/f9gych5t5k/1).

## Abbreviations:

HBS: heme-binding site
HDO: heme-dependent oligomerization
iELISA: indirect ELISA
IMD: indwelling medical device
IPG: Identical Protein Group
MW: molecular weight
pAb: polyclonal antibody
PJI: periprosthetic joint infection
PPIX: protoporphyrin IX
PSU: pilosebaceous unit
SEC: size-exclusion chromatography
sELISA: sandwich ELISA
